# The legacy of past droughts induces water‐sparingly behaviour in Grüner Veltliner grapevines

**DOI:** 10.1111/plb.13620

**Published:** 2024-02-05

**Authors:** J. C. Herrera, S. Savoi, J. Dostal, K. Elezovic, M. Chatzisavva, A. Forneck, T. Savi

**Affiliations:** ^1^ Department of Crop Sciences, Institute of Viticulture and Pomology University of Natural Resources and Life Sciences Vienna Austria; ^2^ Department of Agricultural, Forest and Food Sciences University of Turin Grugliasco Italy; ^3^ Department of Integrative Biology and Biodiversity Research, Institute of Botany University of Natural Resources and Life Sciences Vienna Austria

**Keywords:** drought acclimation, drought adaptation, drought memory, gas exchange, stomatal density, stomatal size, *Vitis vinifera*

## Abstract

- Drought is becoming more frequent and severe in numerous wine‐growing regions. Nevertheless, limited research has examined the legacy of recurrent droughts, focusing on leaf physiology and anatomy over consecutive seasons.
- We investigated drought legacies (after 2 years of drought exposure) in potted grapevines, focusing on stomatal behaviour under well‐watered conditions during the third year. Vines were subjected for two consecutive years to short‐ (SD) or long‐term (LD) seasonal droughts, or well‐watered conditions (WW). In the third year, all plants were grown without water limitation. Water potential and gas exchange were monitored throughout the three seasons, while leaf morpho‐anatomical traits were measured at the end of the third year.
- During droughts (1st and 2nd year), stem water potential of SD and LD plants fell below −1.1 MPa, with a consequent 75% reduction in stomatal conductance (*g*_*s*_) compared to WW. In the 3rd year, when all vines were daily irrigated to soil capacity (midday stem water potential ~ −0.3 MPa), 45% lower values of *g*_*s*_ were observed in the ex‐LD group compared to both ex‐SD and ex‐WW. Reduced midrib vessel diameter, lower leaf theoretical hydraulic conductivity, and smaller stomata were measured in ex‐LD leaves compared to ex‐SD and ex‐WW, likely contributing to the reduced gas exchange.
- Our findings suggest that grapevines exposed to drought may adopt a more water‐conserving strategy in subsequent seasons, irrespective of current soil water availability, with the degree of change influenced by the intensity and duration of past drought events.

## Graphical abstract

Grapevines with a drought history showed increased water use efficiency in a non‐water‐limited season, in coordination with morpho‐anatomical modifications of leaf, xylem anatomy and stomata, suggesting significant drought legacies.

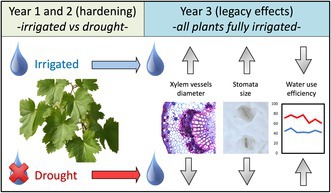

## INTRODUCTION

Grapevines are often exposed to droughts of variable intensity and length during the growing season, particularly in rainfed viticulture. This is the case in most wine growing areas in Europe, characterized by wet springs and dry summers, where drought events are becoming more frequent and extreme (Costa *et al*. 2016; van Leeuwen *et al*. 2019a). Because irrigation is not always a viable solution (e.g. through water scarcity), efforts have focused on investigating genetic tolerance traits of different cultivars, clones or rootstocks (Serra *et al*. 2014; Zhang *et al*. 2016; Dayer *et al*. 2022). Also, different vineyard management strategies have been tested in order to improve water availability for plants (Savi *et al*. 2019b; van Leeuwen *et al*. 2019b). However, data on drought “legacy effects”, grapevine long‐term adaptation strategies and their effects on plant responses to different water availability scenarios are still scarce, especially those addressing the interplay between physiological and anatomical plant traits.

But how do vines remember drought? It is known that drought can promote changes at physiological, morpho‐anatomical, metabolic and epigenetic levels that, in turn, impact plant responses to successive drought events. Recurrent drought can trigger a “priming” effect, often referred to also as “hardening” or “drought memory” (Walter *et al*. 2011; Ding *et al*. 2012; Fleta‐Soriano & Munné‐Bosch 2016). Most previous research explored within‐season drought memory, focusing mainly on annual crops (Wojtyla *et al*. 2020; Liu *et al*. 2022; Sadhukhan *et al*. 2022), while studies addressing the legacy of multi‐season drought stress in perennial crops are scarce. Some attempts have highlighted the great complexity of plant–environment interactions. For example, previous studies on grapevine have shown that if plants survive an intense drought, they will present a more robust response to a later water scarcity (Cramer *et al*. 2007; Lovisolo *et al*. 2010; Tombesi *et al*. 2018; Zamorano *et al*. 2021). Grapevine acclimation and adaptation mechanisms triggered by drought have been examined. Most studies considered only short‐term seasonal mechanisms (i.e. within the year or season of study), including changes in leaf and root anatomy (Cuneo *et al*. 2021), leaf osmoregulation and abscission (Martorell *et al*. 2015; Hochberg *et al*. 2017b; Herrera *et al*. 2022), shifts in vulnerability to embolism (Hochberg *et al*. 2017a; Sorek *et al*. 2021), among others. Studies on long‐term (multi‐season) mechanisms are scarce and often omit hardened plants in a control well‐watered treatment, focusing mostly on drought conditions (Tombesi *et al*. 2018; Zamorano *et al*. 2021). In this context, it has been shown that recurrent droughts lead to a reduction in xylem vessel size in newly formed wood rings of mature stems (Lovisolo & Schubert 1998; Hochberg *et al*. 2017a; Munitz *et al*. 2018; Netzer *et al*. 2019), a feature that potentially influences plant hydraulics, leaf formation, and whole‐plant water relations in the following seasons.

In this study, we examined gas exchange under well‐watered conditions in potted vines that had previously been subjected to 2 years of seasonal drought stress. We hypothesized that past drought hardening will influence plant behaviour during year 3, even if characterized by high water availability. A more water‐spending strategy (i.e. higher transpiration and stomatal conductance) in previously drought‐stressed plants is expected according to Tombesi *et al*. (2018) and Zamorano *et al*. (2021). Finally, we hypothesize a morpho‐anatomical legacy, with a reduction in xylem vessel diameter in leaves of hardened plants.

## MATERIAL AND METHODS

The study was conducted in the experimental site of the University of Natural Resources and Life Sciences Vienna (Tulln an der Donau, Austria) over three consecutive growing seasons (1st, 2nd and 3rd year) using potted grapevines. During the 1st and 2nd year, deficit irrigation treatments were imposed to a subset of plants, while in the 3rd year all plants were equally maintained under fully irrigated conditions (see below).

### Plant material

A total of 36 grapevine plants (*Vitis vinifera* cv. Grüner Veltliner, grafted on Kober 5BB rootstock) were acquired from a local nursery (certified material), potted in 7‐L pots and grown in a glasshouse without water and/or nutrient limitations for one season (year 0). Grüner Veltliner is Austria's most renowned and planted cultivar (>30% of vineyard surface) for white wine production, while the selected rootstock (widely used in European viticulture) can adapt to a wide range of soil types. In early spring of the 1st year, the vines were transplanted into 20‐L pots filled with commercial substrate (CL ED73 Einheitserde, Sinntal, Germany) supplemented with 20% perlite. Thereafter, the plants were positioned outside and randomly arranged in three rows of 14 plants each (i.e. original 36 + 2 “border” vines at the start and end of each row). All pots were positioned on two stapled concrete slabs (each of 50 × 50 × 4 cm) to exclude water absorption from the soil. Rows were north–south oriented with 3.2 m between rows and 1 m between vines. A rain shelter was positioned on each row, similar to that described in Herrera *et al*. (2015) (see Figure S1). This rain shelter (3.8 m high × 2.2 m wide) had a central semi‐circular arch, with a transparent film cover (FVG Euro 4^®^ 180‐μm thick, 89% light transmittance; FVG Folien‐Vertriebs, Dernbach, Germany) and open at the sides to allow air flow and avoid significant changes to the plant microclimate. The vines were pruned to two buds to allow vertical development of two shoots. In the 2nd year, each of the two previously developed canes was pruned to two buds, allowing development of four shoots per plant.

Two irrigation lines were installed per each row, independently controlled by two separated electric valves (AquaPro^®^; Netafim); the first line fed the well‐watered plants (WW, control), while the second was connected to the pots subject to deficit irrigation (SD and LD; see below). In each pot, two pressure‐compensated drippers (2 L·h^−1^ JPI^®^; Netafim, Hatzerim, Israel) were installed, assigning each irrigation line randomly (i.e., irrigation treatments were randomized in each row). In the central row, two balances (PLS 100, max. capacity = 100 kg, sensitivity = 10 g; Meter, München, Germany) were positioned below two plants (one WW, one LD) to continuously monitor plant water consumption. Crop evapotranspiration was thus measured as plant daily weight loss (ET_lys_; calculated as weight loss from 04:00 to 22:00 h; Figure S2) and used to adjust the irrigation volumes for all treatments. All plants were daily irrigated (at midnight) with a water volume equivalent to 120% ET_lys_ until the imposition of deficit irrigation treatments.

### Hardening period: Deficit irrigation treatments (1st–2nd year)

Differentiation of irrigation (hardening) was imposed in early July (DOY 192 and 177, in 1st and 2nd year, respectively) about 45 days after anthesis (DAA; anthesis stage E‐L 23, as defined in Coombe 1995). The plants were divided into three irrigation treatments: (i) well‐watered (WW, control), which continued to receive daily irrigation volumes equivalent to 120% ET_lys_ throughout the entire season; (ii) short deficit irrigation (SD), watered with 35% ET_lys_ of WW for 25 days; and (iii) long water deficit (LD), watered with 35% ET_lys_ of WW for 70 days. Thereafter, the irrigation volumes were re‐imposed at 120% ET_lys_ for all plants.

### Drought legacy under WW conditions (3rd year)

In late winter of the 2nd year, all plants were pruned to two buds and moved from the field into a greenhouse to minimize climate variability and plant damage or stress (e.g. spring frost, hail, heatwaves) that could jeopardize the results. The chamber was set at 24/18 °C (day/night) and 50% relative humidity, supplemented with 12 h (07:00–19:00 h) artificial light from 10 sodium lamps positioned 1.5 m above the plants (PPFD 600 μmol·m^−2^·s^−1^ at origin). Plants were randomly arranged in six rows and daily irrigated to soil capacity (i.e. water dripping from pot base) using the same drip irrigation system described above. No differential irrigation treatments were imposed. Therefore, all plants (from now on named ex‐WW, ex‐SD, ex‐LD) were well‐watered and received the same amount of water every day throughout the 3rd year of the experiment.

### Water relations and gas exchange measurements

Water potential (Ψ) and leaf gas exchange were monitored with a pressure chamber (3000 Series Plant Water Status Consoles; Soilmoisture, Santa Barbara, CA, USA) and a portable gas‐exchange system (LCpro‐S;, ADC BioScientific, Hertfordshire, UK). To assess the imposed level of drought stress, measurements of Ψ_stem_ (Herrera *et al*. 2021) were performed every 7–10 days (only on sunny and clear days) at noon in 3–4 plants per treatment (2 leaves per plant) during the 1st and 2nd year. In the 3rd year, since irrigation volumes were high and equal for all plants we did not expect Ψ to vary during the experiment. Hence, we measured stem and leaf water potential (Ψ_stem_ and Ψ_leaf_, respectively; Savi *et al*. 2019a) only at the end of the vegetation period (70 DAA) in order to rule out eventual differences in water status among treatments. Stomatal conductance to water vapour (*g*_*s*_), leaf transpiration (*E*) and carbon assimilation (*A*) were measured every 4–7 days during the 3 years of the study on 4–7 plants per treatment at each date (1 leaf per plant) using the instruments settings described in Herrera *et al.* (2021).

### Measurements of leaf anatomical traits

At the end of the 3rd year, leaves for morpho‐anatomical measurements were sampled from all study plants. Three mature, fully developed leaves were detached from each potted plant early in the morning. The first leaf was immediately inserted in a falcon tube filled with ethanol 50% and used for analyses of midrib xylem anatomy (see below). The other two leaves were wrapped in cling film, placed in a sealed bag containing wet paper and transported to the laboratory. One was used for leaf mass per unit surface area (LMA) measurements after a 2‐h rehydration, according to Savi *et al*. (2017). Finally, the last sampled leaf was used to obtain negative impressions of the lower leaf epidermis using nail varnish. The impressions were photographed under a light microscope (CX41RF; Olympus, Tokyo, Japan) equipped with an Olympus SC50 digital camera. The stomatal density was calculated by counting stomata in a known area of the imprint, while sttomatal size was measured as maximum length of the aperture using image J (National Institutes of Health, Bethesda, MD, USA). Approximately 50 stomata were measured on two different imprints of each leaf.

For xylem anatomical analyses, a central (about 5‐mm long) portion of the midrib was cut from each leaf. A 20‐μm thick section was cut with a manual rotary microtome (Leica RM2235; Leica Microsystems, Wetzlar, Germany) and stained with Safranin and Astra blue (Carl Roth; Piermattei *et al*. 2015). Slices were then photographed with a Leica CTE6000 microscope equipped with a DMC2900 digital camera. Vessel density, mean vessel diameter and wall thickness were measured using ImageJ on all conduits found in the section. The theoretical hydraulic conductivity (*k*_t_) was calculated according to the Hagen–Poiseuille equation (Tyree & Ewers 1991).

### Statistical analyses

Data were analysed using SPSS version 26.0 (IBM, Armonk, NY, USA). Normality of residuals and homogeneity of variance assumptions were verified. One‐way ANOVA was used to identify differences in traits between experimental treatments; when needed, post‐hoc Tukey's honest significant difference (*P* < 0.05) test was used to separate means. All figures were created using SigmaPlot version 14 (Systat Software, CA, USA). Values are given as mean ± SE.

## RESULTS

### Hardening period: Deficit irrigation treatments (1st–2nd year)

**Fig. 1:**
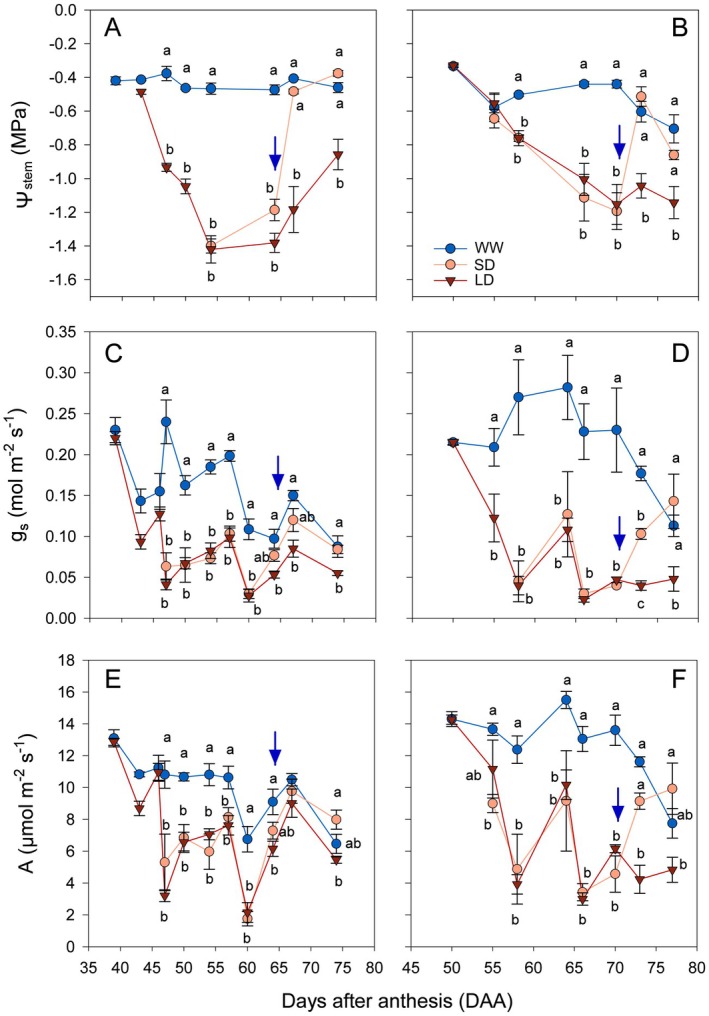
Midday stem water potential (Ψ_stem_; A, B), stomatal conductance to water vapour (*g*_*s*_; C, D), and assimilation rate (A; E, F) of Grüner Veltliner grapevines subjected to well‐watered (WW, blue circles), short‐term (SD, orange circles), or long‐term (LD, red triangles) deficit irrigation in the 1st (A, C, E) and 2nd (B, D, F) year (drought hardening). The blue arrow indicates re‐irrigation in SD treatments. Different letters denote statistically significant differences among treatments for a given day after a Tukey‐HSD test (*P* < 0.05).

As expected, deficit irrigation led to a drop in Ψ_stem_ and leaf gas exchange in all plants and in both years of hardening (Fig. 1A,B). Long and short deficit irrigation (LD and SD, respectively) led to a similar decrease in Ψ_stem_ that reached minimum seasonal values of −1.4 MPa and −1.2 MPa in the 1st and 2nd year, respectively. The Ψ_stem_ of WW controls ranged between −0.45 and −0.50 MPa throughout the experiments. In LD, Ψ_stem_ values remained significantly lower than WW controls until the end of the observation period. On irrigation resumption, Ψ_stem_ of SD plants promptly returned to values not significantly different from those of WW and these values were maintained until the end of the season.

Gas exchange parameters followed similar trends to Ψ_stem_. Briefly, *g*_*s*_ of WW plants ranged between 0.3 and 0.1 mol·m^−2^·s^−1^ during the study seasons. In both years LD and SD plants exhibited rapid stomatal closure following the Ψ_stem_ reduction and reached minimum values of 0.025 mol·m^−2^·s^−1^. The *g*_*s*_ remained <0.1 mol·m^−2^·s^−1^ during most of deficit irrigation treatment, i.e. for 30 and 20 days in LD and SD, respectively. In contrast, *g*_*s*_ in WW plants was 60% higher than in LD and SD during the deficit irrigation period. After resuming full irrigation in SD, *g*_*s*_ values increased >0.1 mol·m^−2^·s^−1^ but were not statistically different from WW (*P* > 0.05; Fig. 1C,D). The trend in transpiration (*E*) reflected that described for *g*_*s*_ (data not shown). Carbon net assimilation (*A*) ranged between 15–7 μmol·m^−2^·s^−1^ under well‐watered conditions but was limited, as expected, by stomatal closure in LD and SD (Fig. 1E,F), with minimum seasonal values of 2–4 μmol·m^−2^·s^−1^.

### Drought legacy under well‐watered conditions

**Fig. 2:**
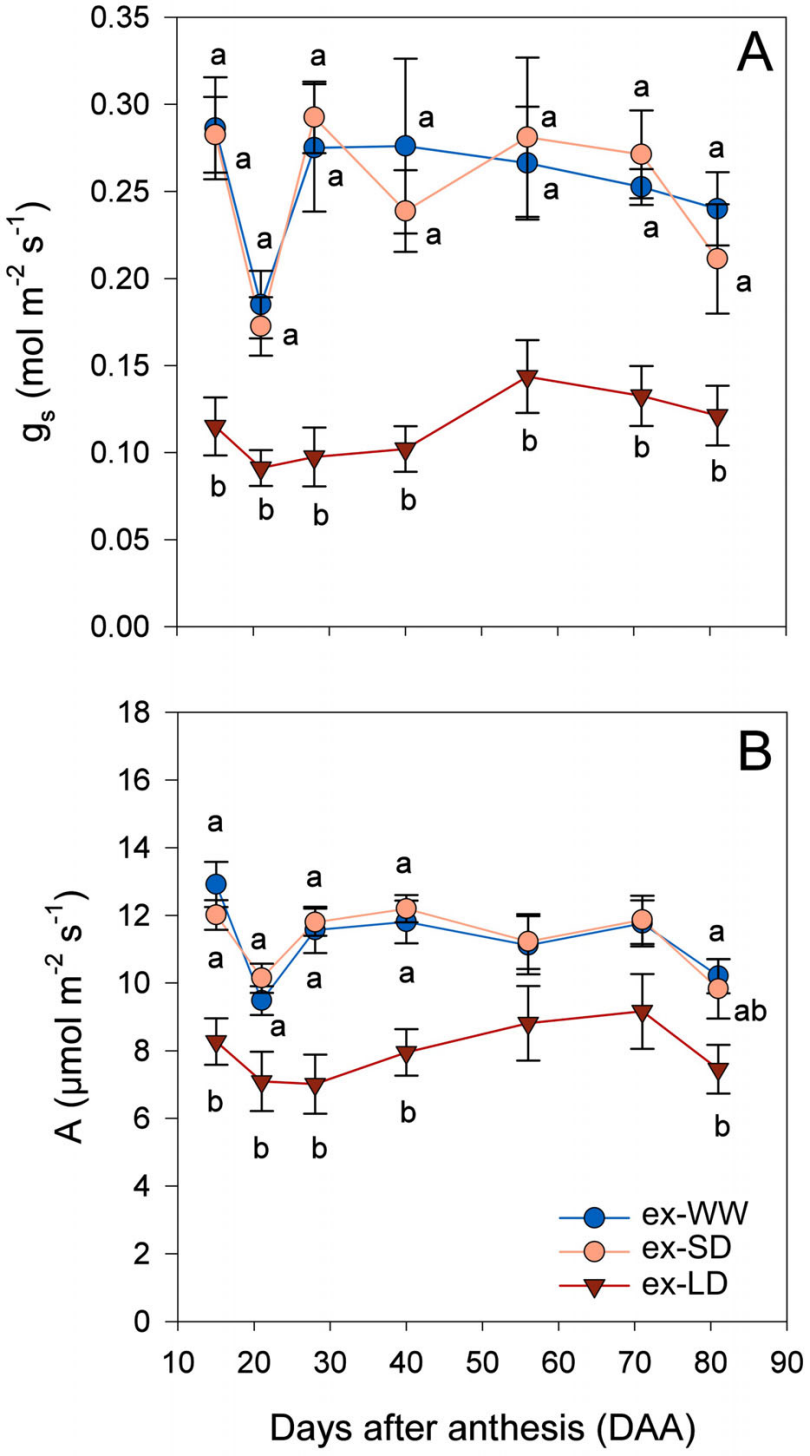
(A) Stomatal conductance to water vapour (*g*_*s*_) and (B) net assimilation rate (*A*) of Grüner Veltliner grapevines in the 3rd year. All plants were daily irrigated to soil capacity. Different letters denote significant differences (*P* < 0.05) after a Tukey‐HSD test among former well‐watered (ex‐WW, blue circles), short‐term (ex‐SD, orange circles), or long‐term (ex‐LD, red triangles) deficit irrigation treatments from the 1st and 2nd year (hardening).

**Fig. 3:**
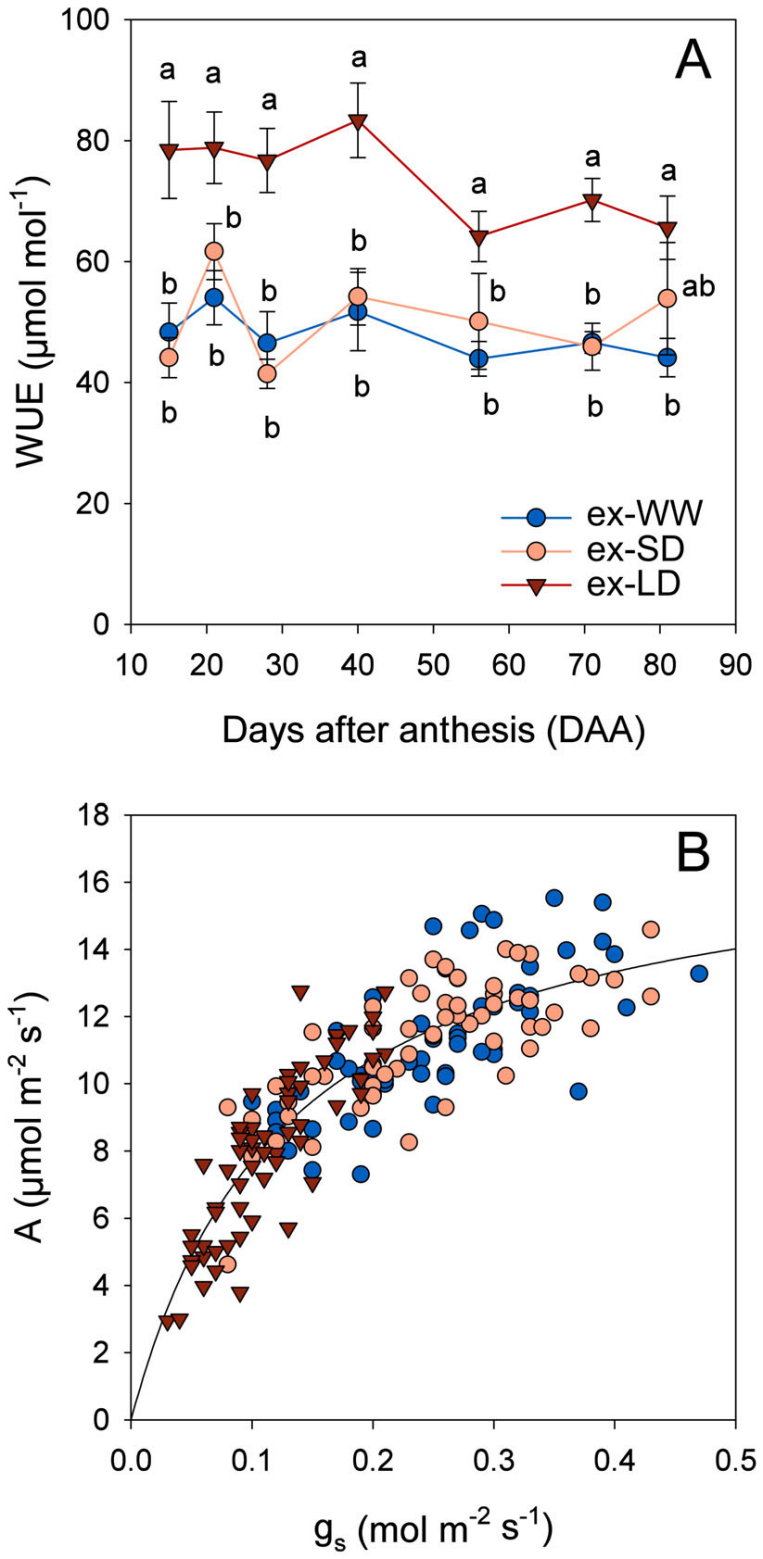
(A) Water use efficiency calculated as the ratio between net carbon assimilation rate (*A*) and stomatal conductance to water vapour (*g*_*s*_) at any given observation date. (B) Relationship between *A* and *g*_*s*_, in Grüner Veltliner grapevines in the 3rd year (high water availability). Different letters denote significant differences (*P* < 0.05) after a Tukey‐HSD test among former well‐watered (ex‐WW, blue circles), short‐term (ex‐SD, orange circles), or long‐term (ex‐LD, red triangles) deficit irrigation treatments from the 1st and 2nd year (hardening).

After two growing seasons of deficit irrigation, throughout the 3rd year of the study all plants were fully irrigated (i.e. all received the same amount of water per day) and leaf gas exchange monitored. Midday leaf Ψ measured 70 DAA was, on average, −0.6 MPa, while average midday Ψ_stem_ was −0.3 MPa, with no differences among the three treatment groups in either case (*P* > 0.05; Figure S3). The ex‐WW and ex‐SD vine *g*_*s*_ values (Fig. 2) ranged between 0.20–0.30 mol·m^−2^·s^−1^ (*E* was 3–4 mmol·m^−2^·s^−1^; Figure S4), while the ex‐LD group maintained *g*_*s*_ between 0.08–0.15 mol·m^−2^·s^−1^ (*E* always <2.5 mmol·m^−2^·s^−1^). During the entire period of observation, *g*_*s*_ and *E* in ex‐LD plants was significantly lower than in ex‐WW and ex‐SD vines (*P* < 0.05), with no differences between the last two groups on any of the measurement days. In other words, vines that had been exposed for two consecutive seasons to long summer water deficits showed a reduction in *g*_*s*_ of about 55% (−40% in *E*) compared to plants that had never suffered from drought or those which received a shorter period of stress (SD). Similarly, carbon assimilation rates of ex‐LD plants were on average 8 μmol·m^−2^·s^−1^ and significantly lower than the average 12 μmol·m^−2^·s^−1^ measured in ex‐WW and ex‐SD for most of the observation period. Therefore, *A* of ex‐LD vines fell by ca. 30% compared to ex‐WW and ex‐SD plants. Consequently, the intrinsic water use efficiency (WUE, ratio between *A* and *g*_*s*_; Fig. 3A) was significantly higher in ex‐LD (average 70 μmol·mol^−1^) than in ex‐WW and ex‐SD (average 45 μmol·mol^−1^). However, the relationship between *g*_*s*_ and *A* showed that all three treatments were distributed along the same curve, albeit ex‐LD never surpassed *g*_*s*_ values of 0.2 mol·m^−2^·s^−1^ whereas ex‐WW and ex‐SD had *g*_*s*_ values up to 0.45 mol·m^−2^·s^−1^ (Fig. 3B).

**Fig. 4:**
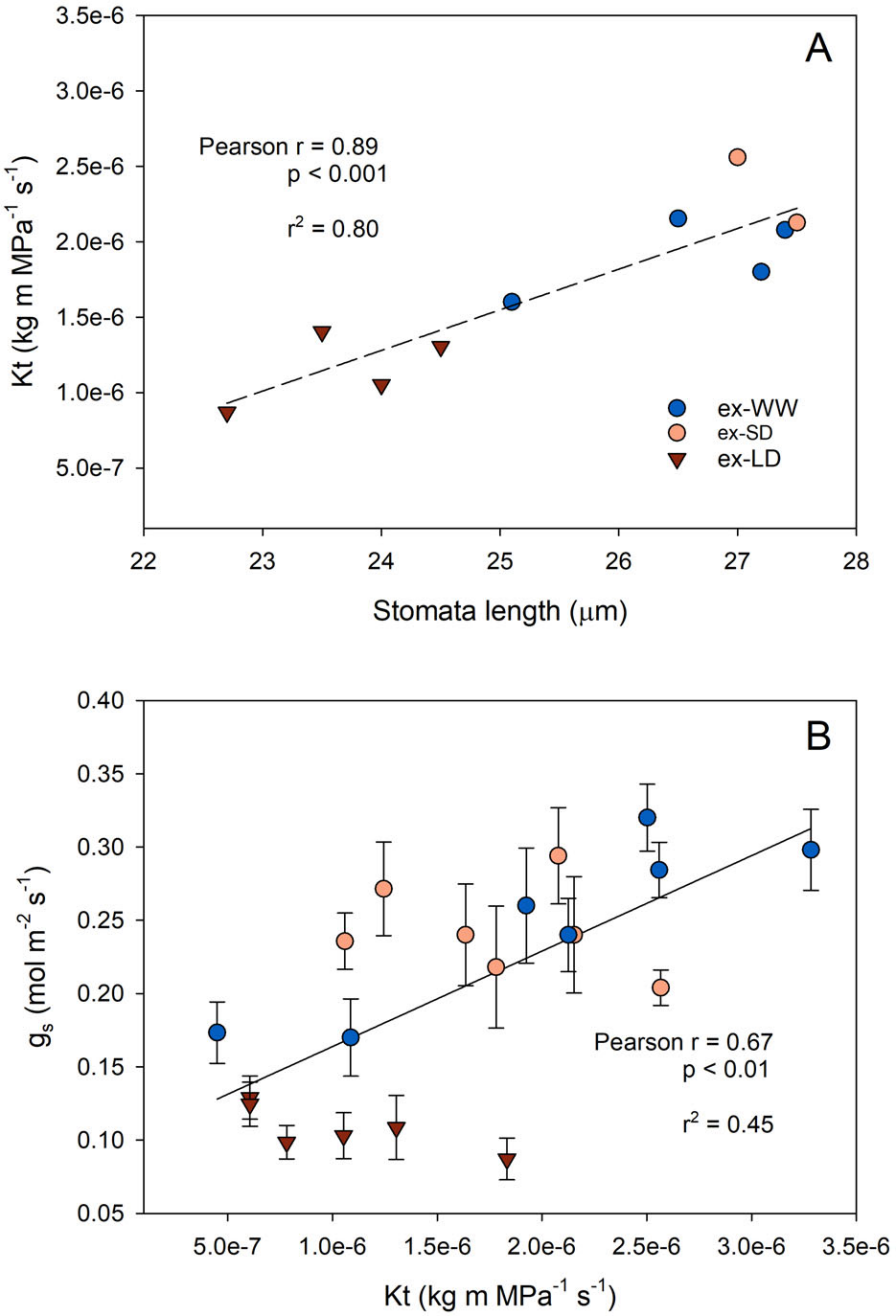
Relationship between (A) theoretical hydraulic conductivity (*k*_t_) of the midrib and average leaf stomata length, and (B) seasonal average stomatal conductance to water vapour (*g*_*s*_) and midrib *k*_t_, from Grüner Veltliner grapevine leaves in the 3rd year (high water availability). Former well‐watered (ex‐WW, blue circles), short‐term (ex‐SD, orange circles), and long‐term (ex‐LD, red triangles) deficit irrigation treatments from the 1st and 2nd year (hardening). In (B) the error bars are SE of the mean (n = 7–10).

The ex‐LD vines developed significantly smaller leaves (−25% leaf area on average) than ex‐WW and ex‐SD vines, albeit the leaf mass per area (LMA) was similar among groups (Table 1). The anatomical analyses showed that the midrib of ex‐LD leaves had a xylem vessel diameter by about 12% smaller (*P* < 0.05) compared to both ex‐WW and ex‐SD, but with a similar vessel density (number of vessels per mm^2^) and cell wall thickness (Table 2; Figure S6). As a result of the differences in xylem anatomy, the calculated theoretical hydraulic conductivity (*k*_t_) was also lower (−45% on average) in ex‐LD compared to ex‐WW and ex‐SD plants. Finally, the leaf stomatal density was not significantly different between treatments (ca. 170 stomata·mm^−2^), but stomata length was significantly smaller (about 11%) in ex‐LD (23.7 μm) compared to ex‐WW and ex‐SD (26.5 and 26.9 μm, respectively). Several leaf physiological and anatomical traits were correlated, for example Fig. 4 shows that an increasing *k*_t_ translated to stomata with larger pore size (*r* ^2^ = 0.80) and, consequently, higher rates of stomatal conductance to water vapour (*r* ^2^ = 0.45).

**Table 1:** Stomatal density and length, average area of a leaf, and leaf mass per area (LMA) of Grüner Veltliner grapevine leaves. Analyses were performed in the 3rd year when all plants were fully irrigated. Former well‐watered (ex‐WW), short‐term (ex‐SD), or long‐term (ex‐LD) deficit irrigation treatments from the 1st and 2nd year (hardening). Values are mean ± standard deviation (n = 4). *ANOVA *P*‐value is reported at the end of each column (in bold when <0.05). Different letters denote significant differences after a post‐hoc Tukey‐HSD test (*P* < 0.05).

	stomatal density (#·mm^−1^)	stomata length (μm)	leaf area (cm^−2^)	LMA (mg·cm^−2^)
ex‐WW	178 ± 28	26.5 ± 1.1 a	258 ± 19 a	4.0 ± 0.5
ex‐SD	156 ± 9	26.9 ± 0.6 a	270 ± 19 a	3.9 ± 0.4
ex‐LD	172 ± 7	23.7 ± 0.8 b	199 ± 17 b	3.8 ± 0.7
*P*‐value*	0.35	**0.002**	**0.030**	0.77

**Table 2:** Midrib xylem anatomy parameters and theoretical hydraulic conductivity (*k*_t_) of Grüner Veltliner grapevine leaves. Leaves were sampled in the 3rd year when all plants were fully irrigated. Former well‐watered (ex‐WW), short‐term (ex‐SD), or long‐term (ex‐LD) deficit irrigation treatments from the 1st and 2nd year (hardening). Values are mean ± standard deviation (n = 8). *ANOVA *P*‐value is reported at the end of each column (in bold when <0.05). Different letters denote significant differences after a post‐hoc Tukey‐HSD test (*P* < 0.05).

	vessel diameter (μm)	vessel density (#·mm^−1^)	cell wall thickness (mm)	*k*_t_ (·10^−6^) (kg·m·MPa^−1^·s^−1^)
ex‐WW	22.7 ± 1.5 a	644 ± 86	2.7 ± 0.2	2.0 ± 0.8
ex‐SD	21.6 ± 2.4 ab	677 ± 119	2.6 ± 0.2	1.8 ± 1.0
ex‐LD	20.2 ± 1.1 b	770 ± 119	2.6 ± 0.3	1.1 ± 0.5
*P*‐value*	**0.032**	0.078	0.87	0.07

## DISCUSSION

Overall, our results show that recurrent or prolonged water deficit events affect morpho‐anatomical traits of vine leaves formed in the following growing season, as well as their gas exchange rates, regardless of soil water availability. The grapevines with a history of drought in the two previous years, in the 3rd well‐watered year showed more water‐sparing behaviour by maintaining lower *g*_*s*_ and *E* and higher WUE. Hence, less water was consumed to support metabolism even if water availability was not limited from budbreak to the end of the season. However, such effects were only observed in the LD group, i.e. when the previous drought stress was maintained for a longer time (70 days instead of 30 days per season). These results suggest that drought duration plays a key role in drought priming, as the drought intensity in our study was similar in SD and LD vines, corresponding to moderate stress (Ψ_stem_ ca. −1.3 MPa; Gambetta *et al*. 2020).

Interestingly, the outcomes of our experiment partially contradict two previous studies of grapevines (Tombesi *et al*. 2018; Zamorano *et al*. 2021), where after one or more years of drought hardening, the plants had higher *g*_*s*_ rates compared to well‐watered controls that had never suffered from water scarcity. However, it should be noted that, unlike our study (where plants were well watered in the 3rd year), both the above‐mentioned studies only explored behaviour of the drought‐hardened plants under dry conditions (i.e. plants with drought and non‐drought history were subjected to a similar drought stress) and report few data points from the period when all plants were well hydrated. Moreover, while the intensity and duration of the applied stress during hardening was similar between our study and that of Tombesi *et al*. (2018) – as well as being on potted plants –, Zamorano *et al*. (2021) reported much more negative Ψ_stem_ (−1.5 to −2.1 MPa) under field conditions. Hence, a direct comparison with the studies of Tombesi *et al*. (2018) and Zamorano *et al*. (2021) is difficult. Our study consistently showed that after two consecutive years of prolonged drought, Grüner Veltliner grapevines altered their water‐use behaviour under a non‐limiting water condition, showing a more conservative use of water (i.e., lower *g*_*s*_ and *E* as compared to former WW plants or even vines exposed to short‐term drought events). Unfortunately, due to the limited number of plants available, we were unable to test their responses also under a drought stress situation as we preferred to maintain a higher number of replicates in well‐watered conditions (n = 12). Even if opposite to the previous trend observed in hardened grapevines under drought (Tombesi *et al*. 2018; Zamorano *et al*. 2021), our results are in agreement with a drought‐avoidance strategy expected in perennial species (Levitt 1980). This opens the discussion towards differences in adaptation mechanisms among grapevine cultivars, species and genotypes. For instance, the domestication/breed origin of grapevine cultivars could be related to xylem anatomy characteristics and vulnerability to cavitation (Pouzoulet *et al*. 2020). Also, it remains unknown what the potential role that rootstocks could have played in our experiment (Kober 5BB), compared to those used by Tombesi *et al*. (2018) (1103 Paulsen) and Zamorano *et al*. (2021) (own‐rooted *V. vinifera*). Different rootstock genotypes have been shown to influence xylem architecture (Santarosa *et al*. 2016) and to confer contrasting drought stress resistance in *V. vinifera* (Zhang *et al*. 2016), but also affect the drought memory in other perennials (Serra *et al*. 2014; Faria‐Silva & Silva 2023). Nevertheless, no study has yet directly assessed the contribution of rootstocks to drought memory in grapevines.

The reduced *g*_*s*_ and *E* rates measured in ex‐LD plants might be partially explained by the morpho‐anatomical modifications observed at leaf level. Ex‐LD plants had smaller leaves than ex‐WW and ex‐SD, despite having the same high water availability for all groups (no differences in Ψ_stem_; Table 1). A similar observation was reported by Zamorano *et al*. (2021). Interestingly, our results also highlight a reduction in xylem vessel diameter of the midrib leading to lower water transport capacity (lower theoretical hydraulic conductivity, *k*_t_) in the ex‐LD leaves and potentially higher resistance to embolism formation (Sorek *et al*. 2021; Hacke *et al*. 2022). In a 4‐year experiment on field‐grown Merlot grapevines, the stem theoretical hydraulic conductivity decreased by ca. 30% in droughted plants as compared to irrigated controls through modifications to the xylem anatomy (Munitz *et al*. 2018). Although we did not analyse xylem anatomy in trunk and stems, we assume a similar variation (i.e. xylem narrowing in LD) occurred in our experiment, consistent with the tip‐to‐base conduits widening (Olson *et al*. 2021). First, these morpho‐anatomical differences observed among experimental treatments suggest that leaf development was likely affected by epigenetic changes induced by the drought imprint. Second, differences in leaf structure might, at least partially, explain the contrasting physiology previously described. More specifically, ex‐LD leaves had 45% lower *k*_t_ compared to ex‐SD and ex‐WW (Table 2), a striking similarity with the 40% and 55% reductions in *E* and *g*_*s*_. Indeed, we found a good correlation between the midrib *k*_t_ and average seasonal *g*_*s*_ (Fig. 4B), in agreement to those frequently reported for leaf hydraulic conductance (*k*_leaf_) and *g*_*s*_ (Ye *et al*. 2021) and the general relationship expected between *g*_*s*_ and root‐to‐leaf hydraulic conductance (Sperry *et al*. 1993). Furthermore, reduced stomata size was also observed in ex‐LD plants, a trait previously associated with reduced maximum stomatal conductance (*g*_max_), enhanced water conservation strategy and higher WUE (Doheny‐Adams *et al*. 2012; Dittberner *et al*. 2018; Bertolino *et al*. 2019; Pitaloka *et al*. 2022), thus supporting results observed in our experiment. Decreased stomatal size has been reported in other plant species grown under low soil moisture (Doheny‐Adams *et al*. 2012; Sun *et al*. 2014) and has been suggested as a drought‐memory trait in European beech (Petrik *et al*. 2022) and potato seedlings (Zhang *et al*. 2018). Even though a low number of replicates were analysed, we found a significant correlation between stomata size and the midrib *k*_t_ (Fig. 4A), as well as with average seasonal *g*_*s*_ (Figure S5). The relationship between stomata and xylem traits has been investigated in some tree species, highlighting a convergent allometric co‐variation of xylem area and stomata size that modulates the balance between water vapour loss through stomata and water delivery via the xylem (Aasamaa *et al*. 2001; Zhong *et al*. 2020). Interestingly, other traits such as the thickness of conduit walls (Table 1) and leaf mass per area (LMA; Table 2) were very similar among ex‐LD, ex‐SD and ex‐WW, suggesting that biomass investment in the leaves was overall similar within a season. In that respect, even if reduced assimilation rates were observed in the 3rd year in LD vines, the rates were comparable to those reported in field‐grown grapevines under deficit irrigation, leading to adequate yield for premium wine grapes (Munitz *et al*. 2020). How drought legacies might impact source–sink ratios, yields and composition of the grape requires further clarification.

Taken together, the plasticity of the phenotype observed in our experiments suggests that coordination of several traits was altered as a legacy of past droughts experienced by the plants. This will require long‐term monitoring of drought legacy effects under both well‐watered and drought‐stressed conditions. Future research should explore the role of epigenetics, as well as the dynamics of non‐structural carbohydrates (NSC) during both hardening period and post‐hardening years. It has been suggested that NSC play a fundamental role during drought (Savi *et al*. 2016; Liu *et al*. 2023; Prats *et al*. 2023), generally resulting in reduced carbohydrate reserves at the end of the season (Dayer *et al*. 2013; Herrera *et al*. 2015). These energy reserves dictate sprouting and growth in the following season, as well as resilience of the plants to future stressors. Moreover, xylem architecture of a wood ring formed under a stressful environment constitutes an important structural legacy in many perennials. In fact, usually only the last two to five annual rings actually conduct water and modulate resistance to water flow from roots to buds. In spring, this hydraulic pipe inexorably influences the amount of water transported, the turgor of growing leaf cells (chlorenchyma, stomata, vascular bundles) and, lastly, the size of xylem conduits in mature shoots and leaves (Tables 1 and 2). Hence trade‐offs between hydraulic conductance, vulnerability to embolism, and building carbon costs are key aspects that also need to be clarified to better understand plant adaptation to drought and the drought legacies in perennial crops.

## CONCLUSIONS

Our experiments shed light on morpho‐anatomical and physiological adaptations in grapevine leaves as a likely consequence of recurrent droughts. Interestingly, only prolonged periods (70 days in this study) of drought stress (Ψ_stem_ = −1.3 MPa, *g*_*s*_ = 0.06 mol·m^−2^·s^−1^) induced alterations in structure and function of the new leaves developed in the following season under high water availability. Such alterations, that we term “drought legacy”, lead to reduced transpiration and photosynthesis through stomatal control. Shorter drought periods of the same intensity apparently did not induce any detectable differences in leaves of the vines compared to those that had never been exposed to drought (Ψ_stem_ > −0.6 MPa, *g_s_* >0.15 mol·m^−2^·s^−1^). We suggest possible mechanisms behind the different leaf physiology observed among treatments, in particular, changes in stomatal size and midrib xylem vessel anatomy, both of which play important roles in leaf hydraulic functioning and plant water relations. Additional grapevine cultivars should be studied, possibly in combination with different rootstocks. Moreover, different drought intensity during hardening and post‐drought growing conditions should also be investigated to understand which cascade effects (yield loss, grape quality, vulnerability to embolism) could be expected in viticulture as a consequence of multiseasons drought legacies.

## AUTHOR CONTRIBUTIONS

JCH and TS conceived and wrote the manuscript. SS, KE (year 1), JD (year 2), and MC (year 3) performed the experiments and acquired the data under the supervision of AF, JCH and TS. All authors read and approved the final manuscript.

## CONFLICT OF INTEREST

The authors declare that they have no known competing financial interests or personal relationships that could influence the work reported in this paper.

## Supporting information


**Figure S1.** Schematic representation of the rain shelter (left) and picture of the experimental site during the Year 1 of experiments.
**Figure S2.** Whole plant daily evapotranspiration (in kg) as measured with the weighing lysimeters, and the daily maximum air vapour pressure deficit (VPD, kPa).
**Figure S3.** Midday leaf and stem water potential measured at 70 DAA in the 3rd year.
**Figure S4.** Leaf transpiration (E) in the 3rd year.
**Figure S5.** Relationship between the mean stomata length and average seasonal stomatal conductance, and the midrib mean vessel diameter and leaf mean stomata length.
**Figure S6.** Midrib cross‐sections of Grüner Veltliner grapevine leaves in the 3rd year.
