# Supplementary material for: The legacy of past droughts induces water‐sparingly behaviour in Grüner Veltliner grapevines
Source: Plant Biol (Stuttg). 2024 Feb 5;28(3):601–9. doi: 10.1111/plb.13620 (PMC13089597; doi:10.1111/plb.13620)
Supplement: Supplementary file 1 — Figure S1. Schematic representation of the rain shelter (left) and picture of the experimental site during the Year 1 of experiments. Figure S2. Whole plant daily evapotranspiration (in kg) as measured with the weighing lysimeters, and the daily maximum air vapour pressure deficit (VPD, kPa). Figure S3. Midday leaf and stem water potential measured at 70 DAA in the 3rd year. Figure S4. Leaf transpiration (E) in the 3rd year. Figure S5. Relationship between the mean stomata length and average seasonal stomatal conductance, and the midrib mean vessel diameter and leaf mean stomata length. Figure S6. Midrib cross‐sections of Grüner Veltliner grapevine leaves in the 3rd year. [file PLB-28-601-s001.docx]

**The legacy of past droughts induces a water-sparingly behaviour in Grüner Veltliner grapevines**

Jose Carlos Herrera^1*^, Stefania Savoi^2^, Josef Dostal^1^, Kristina Elezovic^1^, Martha Chatzisavva^1^, Astrid Forneck^1^, Tadeja Savi^3^

^1^ University of Natural Resources and Life Sciences, Vienna, Department of Crop Sciences, Institute of Viticulture and Pomology, 3430 Tulln an der Donau, Austria.

^2^ University of Turin, Department of Agricultural, Forest and Food Sciences, 10095 Grugliasco, Italy

^3^ University of Natural Resources and Life Sciences, Vienna, Department of Integrative Biology and Biodiversity Research, Institute of Botany, 1180 Vienna, Austria.

**Supporting information**

**Figure S1**. Schematic representation of the rain shelter (left) and picture of the experimental site during the Year 1 of experiments.

**Figure S2**. Whole plant daily evapotranspiration (in kg) as measured with the weighing lysimeters for well-watered (ET_lys WW) and long-term deficit irrigated (ET_lys LD) Grüner Veltliner grapevines in the 1^st^ and 2^nd^ year of experiments (hardening). The daily maximum air vapour pressure deficit (VPD, kPa) as measured by the nearby weather station (2m high and positioned 150 m apart from the experimental site) is also presented.


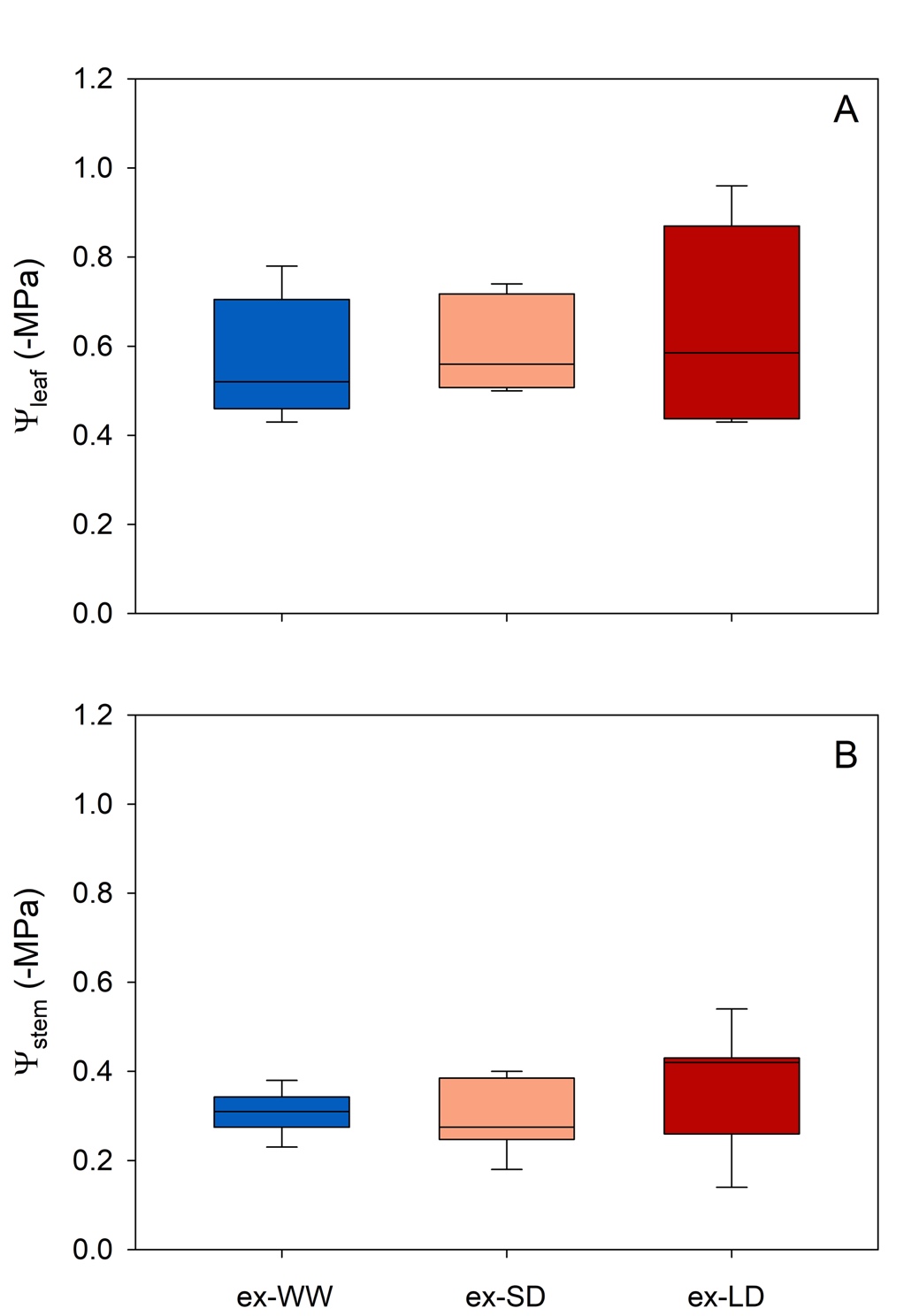


**Figure S3**. Midday leaf (Ψ_leaf_) and stem (Ψ_stem_) water potential of Grüner Veltliner grapevines measured at 70 DAA in the 3^rd^ year. All plants were daily irrigated to soil capacity. No differences (p>0.50) after ANOVA test were observed between former well-watered (ex-WW), short-term (ex-SD), and long-term (ex-LD) deficit irrigation treatments from the 1^st^ and 2^nd^ year (hardening).


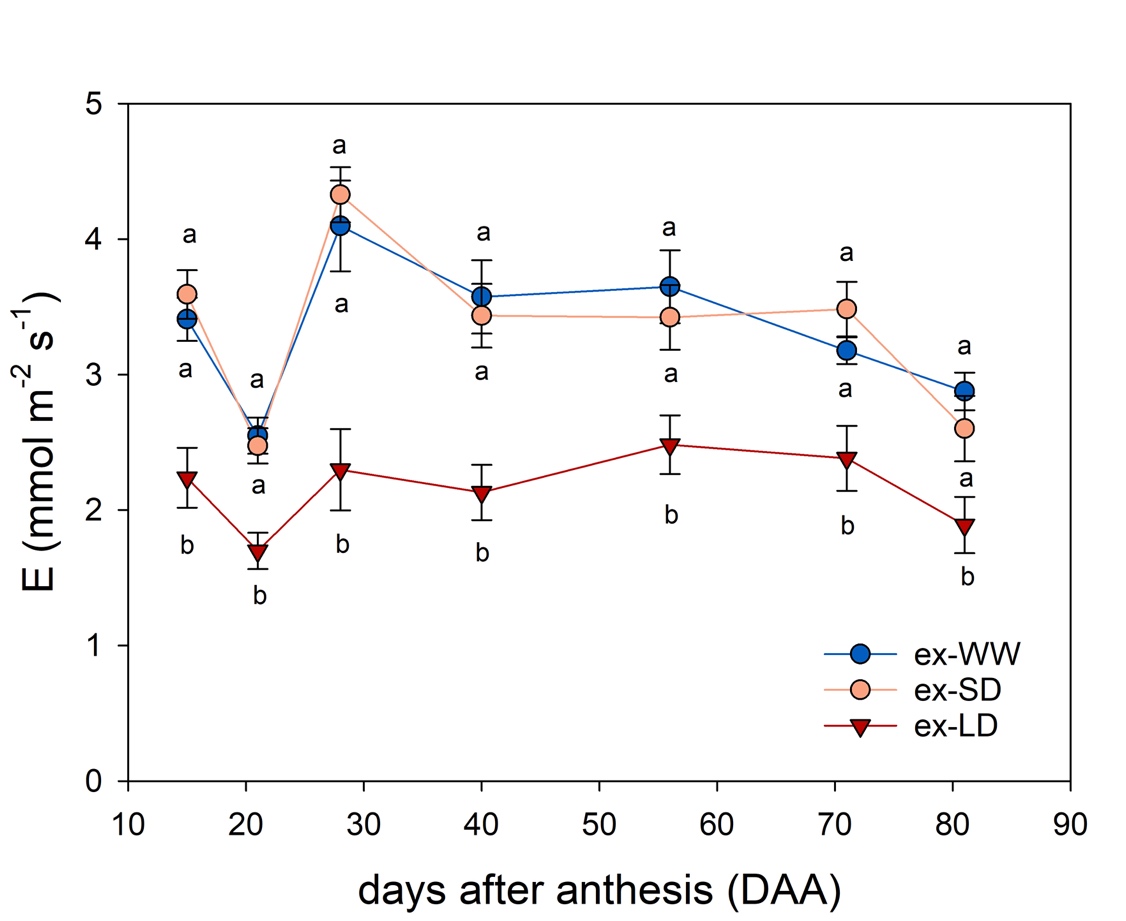


**Figure S4**. Leaf transpiration (E) of Grüner Veltliner grapevines in the 3^rd^ year. All plants were daily irrigated to soil capacity. Different letters denote significant differences (p<0.05) after a Tukey-HSD test among former well-watered (ex-WW, blue circles), short-term (ex-SD, orange circles), or long-term (ex-LD, red triangles) deficit irrigation treatments from the 1^st^ and 2^nd^ year (hardening).

**
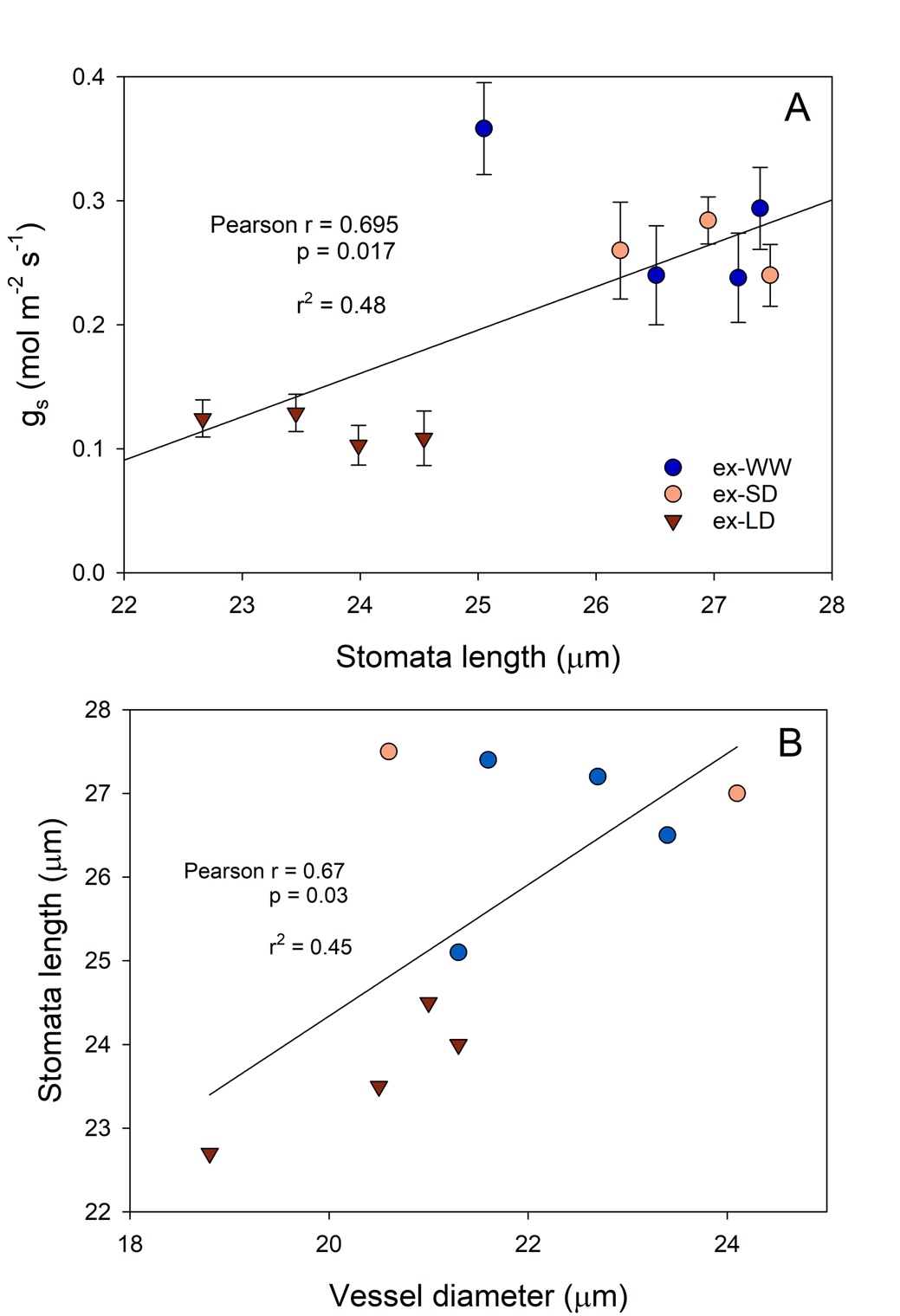
**

**Figure S5**. Relationship between A) the mean stomata length and average seasonal stomatal conductance, and B) the midrib mean vessel diameter and leaf mean stomata length in Grüner Veltliner grapevine leaves in the 3^rd^ year (high water availability). Former well-watered (ex-WW, blue circles), short-term (ex-SD, orange circles), and long-term (ex-LD, red triangles) deficit irrigation treatments from the 1^st^ and 2^nd^ year (hardening)

**Figure S6**. Midrib cross-sections of Grüner Veltliner grapevine leaves in the 3^rd^ year (high water availability). A) former well-watered (ex-WW) and B) former long-term deficit irrigation (ex-LD)
